# Uterine artery embolization for primary postpartum hemorrhage

**Published:** 2013-06

**Authors:** Tae-Hee Kim, Hae-Hyeog Lee, Jun-Mo Kim, Ae-Li Ryu, Soo-Ho Chung, Woo Seok Lee

**Affiliations:** 1 *Department of Obstetrics and Gynecology, College of Medicine, Soonchunhyang University, Bucheon, Republic of Korea.*; 2 *Department of Urology, College of Medicine, Soonchunhyang University, Bucheon, Republic of Korea.*

**Keywords:** *Uterine artery embolization*, *Postpartum hemorrhage*, *Pregnancy*

## Abstract

**Background: **Postpartum hemorrhage is the leading cause of severe maternal morbidity and death. A prompt management of uterine artery embolization (UAE) is important for a good outcome. UAE is generally accepted to be a safe and reliable procedure.

**Objective:** To estimate critical patient characteristics influencing the success of UAE for the treatment of emergent primary postpartum hemorrhage.

**Materials and Methods: **This was a cross sectional study that reviewed 121 patients who were diagnosed primary postpartum hemorrhage between February 2002 and December 2009 at a tertiary treatment center among 4,022 deliveries. We evaluated patient clinical characteristics associated with a successful surgical outcome of UAE.

**Results:** The success rate for UAE was 96%. For two cases, UAE complication was associated with fever (>38.5^o^C). Five patients had problems that required admission to the intensive care unit (ICU).

**Conclusion:** To increase the surgical success rate and lower the number of ICU admissions, the decision to treat primary postpartum hemorrhage using UAE should be based on individual patient clinical findings under the direction of obstetrics staff and an interventional radiologist.

## Introduction

Postpartum hemorrhage is the most serious complication encountered by obstetricians during routine patient care and is the leading cause of severe maternal morbidity and death. The incidence of maternal mortality due to postpartum bleeding varies between countries (1). In developing countries, the incidence of maternal mortality is approximately 1 in 1,000 deliveries, whereas in developed countries, the incidence is only around 1 in 10,0000 deliveries (2). 

This large difference in maternal mortality is primarily attributed to country-specific differences in management capacity. Recommended procedures for management of postpartum hemorrhage have been well published, with recent reports focusing on use of conservative management rather than cesarean hysterectomy (CH) to preserve the uterus. 

The first step in common management of postpartum hemorrhage is the use of uterine stimulants (uterotonics) such as oxytocin, ergot derivatives, prostaglandins, and misoprostol, and bimanual compression of the uterus. Recommended operative procedures for the management of postpartum hemorrhage include surgical repair of lower genital tract lacerations, uterine hypogastric artery ligation, and hysterectomy. 

More recently, the relative benefits of uterine artery embolization (UAE) versus CH have been debated. UAE is generally accepted to be a safe and reliable procedure; however, the success rates and complications for this procedure have been published, and these presented only a small number of cases. Primary postpartum hemorrhage occurs within the first 24 hours following delivery (3). We estimate critical patient characteristics influencing the success of UAE for the treatment of emergent primary postpartum hemorrhage.

## Materials and methods

After obtaining approval from the Institutional Review Board, a retrospective review of hospital records was performed at Soonchunhyang University Bucheon Hospital, a tertiary referral center for primary facilities. Among 4,022 deliveries, One hundred twenty four patients who gave birth in this hospital or who were referred from primary facilities were diagnosed with postpartum bleeding between February 2002 and December 2009. Primary postpartum hemorrhage is defined as blood loss of 500 ml or more as measured by the pad count in the first 24 hours following delivery.

Common blood loss volumes are approximately 500 ml following vaginal delivery and 1,000 ml following Cesarean section. Following three patients were excluded in this study. Two patients were referred to the emergency room for primary postpartum bleeding and died prior to surgery from hypovolemic shock. One patient underwent UAE for secondary postpartum hemorrhage. 

Soonchunhyang University Bucheon Hospital is a bloodless medical center serving Jehovah´s Witnesses. All patients were treated with routine medical management including administration of uterotonics such as oxytocin, ergot derivatives, prostaglandins, and with bimanual massage. Patients were monitored with continuous pulse oxymetry and assessment of heart rate, blood pressure (BP), and urine output thorough an indwelling catheter. Two large-bore intravenous cannulae and a central venous catheter were inserted, and immediate rapid fluid replacement was performed using crystalloids, Ringer`s lactate, or Hartmann`s solution. Blood and coagulation factor transfusion was also performed for every patient excluding Jehovah´s Witnesses. Hospital obstetrics staff performed physical and pelvic examinations on every patient referred from a primary care facility. 

First, an interventional radiologist who can perform UAE at any time is on call. Second, the obstetrics staff and interventional radiologist select the appropriate operative procedure-UAE or CH-based on the patient’s clinical condition and a refractory response to conservative medical management. UAE is preferred for patients who have a stable systolic and diastolic BP or heart rate. UAE is the first-line treatment for postpartum bleeding.

Selective UAE was performed by one of two interventional radiologists. The first interventional radiologist performed procedures from February 2002 through August 2006, and the second interventional radiologist performed procedures from September 2006 through December 2009. Using a microcatheter, selective UAE was performed using gelfoam pieces approximately 4 mm in diameter, which provided temporary artery occlusion. Approximately 4-6 weeks after this procedure, the vascular bed recanalizes, and blood flow returns to normal based on Doppler ultrasonography. When bleeding continued after embolization or hysterectomy or when patient vital signs (BP, pulse rate, oligouria) were unstable, the procedure was deemed ineffective, and the alternate surgical procedure was performed in order to stop the bleeding. 

The primary endpoint was the result of the UAE with no other procedure, such as a CH. The secondary endpoint was assessment of the hemoglobin (Hb), hematocrit (Hct), disseminated intravascular coagulation (DIC), diastolic and systolic pressures, pulse, total blood loss, and amount transfused. The patient clinical characteristics of gravity, parity, age, delivery mode, cause of postpartum hemorrhage, and other medical disease (hypertension, diabetics’ mellitus and thyroid disease) were also assessed. Intensive care unit (ICU) admission, days in hospital, need for an additional operation, complications following UAE, comorbidity (other medical disease), time from delivery until UAE, and mortality data were also recorded.

**Statistical analysis**

Mean age, operation time, total blood transfusion, hemoglobin systolic and diastolic BP, heart rate in the two groups were compared using an independent-samples t-test with SPSS ver. 12 (SPSS Inc., Chicago, IL, USA). The Chi-square test was used to examine differences in presence of DIC, expire, Jehovah's Witness, Refer, comorbidity, vaginal delivery in the two groups. 

## Results

**Patients**

From February 2002 through December 2009, 124 patients who gave birth at Soonchunhyang University Bucheon Hospital or who were referred from a primary care facility were diagnosed with postpartum hemorrhage. Three patients who did not undergo UAE or CH within 24 hours after delivery were excluded from further analyses. Among the remaining 121 patients with primary postpartum bleeding, the mean patent age was 31.3±4.2 years, and the mean parity was 2.5±0.2 children; 39 patients were primiparous. The mean duration of gestation was 36.6±2.5 weeks, with 29 (23%) preterm deliveries (<37 weeks). Of these births, 56 were vaginal deliveries and 65 were deliveries by cesarean section. 

The causes of postpartum bleeding were total placenta previa with placenta accreta (4/121, 3.3%); total placenta previa without placenta accreta (4/121, 3.3%); uterine atony (101/121, 83.4%); and vaginal wall laceration (12/121, 9.8%). Sixty-one patients underwent hysterectomy, and 60 patients were treated with UAE. The overall patient mortality rate was 4% (5/121). 

Eight study patients self-identified as Jehovah´s Witnesses (6.6%), one of whom died after refusing blood transfusion, and one of whom died of acute myeloid leukemia. She was diagnosed with acute myeloid leukemia during her pregnancy and died 3 days after the UAE. Three additional patients died as a result of hypovolemic shock, DIC, or acute renal failure.

**Patients treated by UAE**

The mean age of patients treated by UAE was 31.0±4.8 years. This group included 17 primiparous pregnancies and 14 preterm deliveries. Twenty three patients had vaginal deliveries. Five women gave birth to twins, all by cesarean section. The causes of postpartum bleeding were placental previa without accrete (4/60, 7.5%); uterine atony (55/60, 92.4%); and vaginal wall laceration (1/60, 1.6%). 

All patients were administered uterotonic drugs, with 60 (100%) patients receiving oxytocin, 41 (68%) receiving sulprostone (Nalador), and 22 (36%) patients receiving Ervin. Systolic BP or diastolic BP and heart rate immediately before UAE were 127±23.8 (mmHg), 76.4±21.2 (mmHg) and 90.3±17.9 respectively. Patients experienced a mean blood loss of 676.7 ml prior to UAE, with 25/60 (41.6%) patients requiring a blood transfusion. DIC was observed in 4/60 (6.6%) patients. The mean volume of blood transfusion, excluding patients who self-identified as Jehovah´s Witnesses, was 6.1±8.2 pints. 

The mean length of time in the ICU was five days (8.3%), and the mean length of overall hospital stay was 8.60 days. The first interventional radiologist performed 39/60 procedures (65%). The most frequent embolized vessel was the uterine artery, with bilateral UAE performed for 49 patients (81.6%). 

Of the 60 patients who underwent UAE, 22 patients were referred from primary facilities for postpartum hemorrhage. The success rate of UAE was 96%. Two patients who received UAE also underwent subsequent CH, 11 patients had a transient fever (>38.5^o^C) which resolved within two days, no patients exhibited signs of infection in blood culture findings, and no other complications were reported, with the exception of one ovarian failure after UAE. 

However, this patient had a previous history of pelvic arterial embolization as a result of adenomyosis and uterine multiple myomas. She also had a history of infertility and had conceived the present pregnancy through in vitro fertilization. Two years after the delivery, she complained of amenorrhea and hot flashes, symptoms of menopause. We checked FSH and E_2_, and she was diagnosed with premature ovarian failure. 

Four patients with total placenta previa without accrete underwent simultaneous cesarean section and bilateral uterine artery ligation with bilateral tubo-ovarian vessel ligation, but these procedures did not successfully interrupt continued bleeding. In only two patients in the present cohort, UAE fail to control postpartum hemorrhage. For one patient, bleeding arose from the cesarean section uterine wound. Ultrasonography revealed a 10x15x20 cm hematoma in the pelvic cavity. 

Another patient had combined bleeding, and the focus was on the uterine atony and vaginal laceration. Vaginal bleeding continued despite embolization. Vaginal bleeding arose from vaginal and cervical lacerations following vaginal delivery. One patient who self-identified as a Jehovah´s Witness died of acute myeloid leukemia after embolization. No vascular abnormalities were identified during angiography.

**Admission to the ICU after UAE**

The mean length of time in the ICU was 5 days. After UAE, those five patients had problems that required admission to the ICU. The first patient was a Jehovah’s Witness and was diagnosed with preeclampsia with uncontrolled BP. The second patient, who also self-identified as a Jehovah’s Witness, died of acute myeloid leukaemia, which was diagnosed before delivery. The third patient was diagnosed with HELLP syndrome, and the fourth had placenta percreta. The fifth patient complained of chest discomfort and dyspnoea after delivery, and she was suspected of having pulmonary embolism. These five patients had controlled vaginal bleeding after UAE, but each required admission to the ICU. 

**Patients treated by CH**

Of the 61 patients who underwent CH, 38 were referred from local clinics. The success rate of CH was 93%, and four patients initially treated using CH underwent subsequent UAE. The mean age for this cohort was 31.8±4.0 years. Twenty-two women were primiparous. Of these deliveries, 15 were (24.5%) preterm, 33 (54.0%) were vaginal deliveries, and four were twin pregnancies, all of whom underwent cesarean section. The causes of postpartum bleeding were placenta previa with placenta accreta (4/61, 6.5%), uterine atony (46/61, 75.4%), and vaginal wall laceration (11/61, 18.0%). Two patients suffering from placenta with total previa with accrete underwent CH, and two total placenta previa with placenta accreta who were referred from primary facilities underwent CH. 

All patients were administered uterotonic drugs, with 60 (100%) patients receiving oxytocin, 37 (60.6%) receiving sulprostone, and 12 (19.6%) patients receiving Ervin. In total, 36 (59.0%) and 57 (93.4%) patients received a transfusion before and after hysterectomy, respectively. DIC was observed in 34 (55.7%) patients. Patients had a mean blood loss of 1288.3 ml immediately before CH. Total mean blood loss between delivery and CH was 1769.1 ml. The mean number of blood transfusion unit per patient was 6.7 pints, with the exception of Jehovah´s Witnesses, who refused transfusion. Thirty-nine (63.9%) patients were admitted to the ICU, and the mean length of overall hospital stay was 11.5 days. 

Surgical complications included 14 patients with a transient fever (>38.5^o^C) that resolved within 2 days and two patients with skin wound in CH revision. No infections were identified in blood culture findings. For four patients, CH failed to control bleeding. In two patients, arterial hemorrhage arose from extrauterine sites of vaginal bleeding. In the other cases, uterine collateral vessels bled postoperatively despite ligation of the uterine arteries. Bleeding may have resulted from all pelvic vessels being engorged and friable in the postpartum state. Thus, four patients underwent UAE immediately following CH.

**Difference between UAE and CH**

The patient characteristics of age, gestational time, number of previous abortions, newborn weight, and time from delivery to surgical intervention were not significantly different between patients treated with UAE and those treated with CH. However, significant group differences were noted for the variables of DIC, diastolic pressure, systolic pressure, heart rate, total blood loss immediately before surgical intervention (UAE or CH), and total transfusion units (Table I, II).

**Table I:** Clinical characteristics influence the success

	**Uterine Artery Embolization (n=60)**	**Cesarean Hysterectomy (n=61)**	**p-value**
Time (minutes)	249.8 ± 222.2	241.6 ± 123.5	0.813
Total blood transfusion (pint)	6.1 ± 8.2	34.1 ± 47.8	<0.001
Hemoglobin (g/dL)	10.5 ± 2.3	9.0 ± 2.8	0.004
Systolic BP (mmHg)	127 ± 23.8	94.7 ± 31.3	<0.001
Diastolic BP (mmHg)	76.4 ± 21.2	58.4 ± 19.4	<0.001
Heart rate	90.3 ± 17.9	106.8 ± 25.9	<0.001
Age	31.0 ± 4.8	31.8 ± 4.0	0.358

BP: Blood pressure.

p-value : t-test

Time: Time from delivery to surgical intervention.

**Table II:** Clinical characteristics influence the success

	**Uterine Artery Embolization (n=60)**	**Cesarean Hysterectomy (n=61)**	**p-value**
DIC	4 (6.6%)	34 (55.7%)	<0.001
Expire	1 (1.6%)	4 (6.6%)	0.370
Jehovah’s Witness	4 (6.6%)	4 (6.5%)	1.000
Refer	22 (36.6%)	38 (62.2%)	<0.001
Co morbidity	8 (13.3%)	5 (8.1%)	0.376
Vaginal delivery	23 (38.3%)	33 (54.1%)	0.081

DIC: Disseminated intravascular coagulation.

p-value: chi-square test

## Discussion

This report represents the largest cohort of patients with primary postpartum hemorrhage in one hospital and a long-term period of UAE. Primary postpartum haemorrhage is an emergency condition; thus, obstetricians and interventional radiologists should consider the results reported here to help determine the appropriate intervention and increase the success rate of UAE. To increase the surgical success rate and lower the number of ICU admissions, we constructed the following algorithm for the early management of primary postpartum haemorrhage (2). 

If a primary care facility does not have the necessary equipment to treat primary postpartum haemorrhage, patient transfer should be considered as soon as possible (4). An on-call team of obstetricians and interventional radiologists is crucial to increase the success rate of UAE (5). UAE is preferable for patients who have stable systolic BP, diastolic BP, or heart rate. Obstetricians and interventional radiologists should decide whether to perform UAE or hysterectomy based on the above clinical findings (6). A skilled intervention radiologist is necessary to increase UAE success rate (7). 

Following the procedure, we reconsidered whether to admit patients to the ICU based on clinical severity. The primary cause of postpartum hemorrhage in the present patient cohort was uterine atony bleeding. Other causes included placenta abnormality, genital tract laceration, and uterine inversion (2). The reported incidence of CH ranges from 0.2-2.3/1000 deliveries, with substantial variation depending on the reporting country (4). This incidence suggests that CH is currently not the primary intervention utilized to treat postpartum bleeding. In developing countries, primary medical management approaches, such as crystalloid fluid replacement, uterotonics, and blood transfusion, are often not accessible (5). 

In these settings, the purpose of intervention is to preserve the mother’s life and not to rescue the uterus. In developed countries, UAE has been utilized as an alternative method for management of intractable bleeding following failure of medical management, and uterine artery pseudoaneurysm or arteriovenous malformation (6, 7). Since the first report of the successful use of UAE for the treatment of postpartum hemorrhage in 1979, success rates have been infrequently reported (8). These limited reported data include a 100% success rate in a series of nine cases, 15 of 16 successful UAE surgeries with 12 of 16 patients suffering from primary postpartum hemorrhage, and seven of eight successful surgeries in another case series (9-11).

In one report, only five successful cases of UAE were reported over a period of 5 years, with the authors stating that patient transfer for UAE was challenging and that no exact data were available supporting predictors of successful outcomes (12). The UAE success rate in patients with severe hemorrhage (bleeding loss >1500 ml), DIC, and hemodynamic shock was 71.5%, with hemodynamic shock representing a failure factor (13). UAE has been recommended for hemodynamically stable patients with vaginal laceration and uterine atony, but only nine patients treated with UAE were reported, and the authors ultimately recommended bilateral hypogastric artery ligation as a more cost effective treatment than UAE (14). 

If postpartum blood loss is more than 1500 ml, women usually develop hypotension, with a BP below 80 mmHg. In hemodynamically stable patients, UAE is reported to result in successful outcomes. However, the authors did not report detailed findings defining hemodynamic stability (14). UAE had a success rate of 80% in patients with severe bleeding and a mean blood loss of 8000 ml, but only 22 cases were reported, and prophylactic catheterization was performed for all patients (15). Prophylactic catheterization is not possible for patients undergoing transfer to another care facility. In a descriptive article on UAE and CH, the authors emphasized that if a patient is generally stable and interventional radiologist is present, UAE is a safe first-line treatment for postpartum bleeding. 

However, the report did not provide a detailed definition of hemodynamic stability, and only 12 cases of embolization and six cases of CH were evaluated (16). Placenta accreta was one of the major conditions requiring CH following UAE (60% embolization failure) (17-19), but only a small number of cases were reported with no statistical analyses. Although we evaluated only a limited number of patients with this condition, based on the present findings and other reported data, UAE should be cautiously considered for patients with placenta previa with accreta. Rare complications have been reported, including embolus from the site of re-embolization into the femoral artery, which required immediate intervention, and vesicovaginal fistula (20). 

We found no serious complication stemming from the use of gelfoam, possibly because the same gelfoam was used by the same interventional radiologist for all reported patients for same periods. Clinical findings in primary postpartum hemorrhage are the most important predictors of successful UAE. In the present study, the success rate of embolization was 96%. These results may have stemmed from the involvement of only two interventional radiologists in all reported procedures, versus four or seven different interventional radiologists in studies reported previously (21). Thus, the use of the same technique may have been a primary contributing factor to our higher success rate. The present study had two main limitations. The data were retrospective cohort data and the same patient management was not performed across all patients. 

Although the data set contained this bias, all data were manually reviewed by a single obstetrician who confirmed that every patient was initially managed using uterotonics, massage on uterine fundus, or intravenous administration of crystalloid or colloid substances. Another study limitation involved the difficulties encountered in the evaluation of total blood loss for transferred patients. These data were assessed based on physician notes and pad counts from patient records at the primary care facility. Thus, blood loss for transferred patients was an estimate. 

If an obstetrician encounters intractable postpartum bleeding refractory to primary medical management, the present report provides an educational guide for rational selection of UAE or CH. If a primary care facility does not have the necessary equipment for treating primary postpartum hemorrhage, patient transfer should be considered as soon as possible. UAE is a safe and effective procedure for preserving patient fertility following primary postpartum hemorrhage and a team approach involving both obstetricians and interventional radiologists is critically important to increasing the success rate of UAE. 

Our data were obtained from two interventional radiologists, so they may be biased. However, the two interventional radiologists have been performing UAE for more than 10 years. We reviewed their methods for UAE, and both interventional radiologists used the same techniques. Future randomized, controlled trials are required for identification of objective factors predictive of successful application of UAE for the treatment of primary postpartum hemorrhage. 

## Conflict of interest

The authors did not report any potential conflicts of interest.
